# HJH-1, a Broad-Spectrum Antimicrobial Activity and Low Cytotoxicity Antimicrobial Peptide

**DOI:** 10.3390/molecules23082026

**Published:** 2018-08-14

**Authors:** Qing Wang, Yanzhao Xu, Mengmeng Dong, Bolin Hang, Yawei Sun, Lei Wang, Yongqiang Wang, Jianhe Hu, Wenju Zhang

**Affiliations:** 1 College of Animal Science and Technology, Shihezi University, Shihezi 832003, China; wangqing@hist.edu.cn (Q.W.); wangyongqiang@hist.edu.cn (Y.W.); 2 College of Animal Science and Veterinary Medicine, Henan Institute of Science and Technology, Xinxiang 453003, China; xuyanzhao@hist.edu.cn (Y.X.); xxdmm1205@163.com (M.D.); yzhbl001@126.com (B.H.); sunyawei1975@126.com (Y.S.); wanglei_208@yeah.net (L.W.)

**Keywords:** antimicrobial peptides (AMPs), bovine hemoglobin, membrane activity, bioactivity, pore formation

## Abstract

With the overuse of antibiotics, multidrug-resistant bacteria pose a significant threat to human health. Antimicrobial peptides (AMPs) are a promising alternative to conventional antibiotics. This study examines the antimicrobial and membrane activity of HJH-1, a cationic peptide derived from the hemoglobin α-subunit of bovine erythrocytes P3. HJH-1 shows potent antimicrobial activity against different bacterial species associated with infection and causes weaker hemolysis of erythrocytes, at least five times the minimum inhibitory concentration (MIC). HJH-1 has good stability to tolerance temperature, pH value, and ionic strength. The anionic membrane potential probe *bis*-(1,3-dibutylbarbituric acid) trimethine oxonol [DiBAC_4_(3)] and propidium iodide are used as indicators of membrane integrity. In the presence of HJH-1 (1× MIC), *Escherichia* *coli* membranes rapidly depolarise, whereas red blood cells show gradual hyperpolarisation. Scanning electron microscopy and transmission electron micrographs show that HJH-1 (1× MIC) damaged the membranes of *Escherichia coli*, *Staphylococcus aureus*, and *Candida albicans*. In conclusion, HJH-1 damages the integrity of the bacterial membrane, preventing the growth of bacteria. HJH-1 has broad-spectrum antibacterial activity, and these activities are performed by changing the normal cell transmembrane potential and disrupting the integrity of the bacterial membrane.

## 1. Introduction

The emergence of antibiotic-resistant strains of bacteria is a serious threat to public health, such as the appearance of bacteria with New Delhi metallo-β-lactamase (NDM-1) [1,2]. Such bacteria are spreading in communities throughout the world. More seriously, bacteria can exchange drug-resistance genes [3,4]. Traditional antibiotics kill bacteria by disturbing or blocking their normal metabolic processes. However, by modifying their genetic structure and the antibiotic target site, bacteria can avoid or resist antibiotics. Therefore, there is an urgent need to develop innovative therapies to treat infections caused by multidrug-resistant bacteria. With their distinct mechanism of action against a spectrum of bacteria and viruses, including multidrug-resistant bacteria and human immunodeficiency virus (HIV) [5], antimicrobial peptides (AMPs) are potential important therapeutic agents [6].

AMPs are components of the innate immune system. With the isolation and characterisation of novel AMPs from a variety of organisms and tissues, researchers have examined the relationship between the structure and activity of AMPs [7,8]. Although their mode of action is not fully understood, it is generally known that they target the cytoplasmic membrane of bacteria. Most AMPs are positively charged, whereas the lipid bilayers of bacteria are negatively charged. Consequently, the AMPs adsorb onto the lipid bilayers via electrostatic interactions. Oligomers of the AMPs can insert themselves into the membrane, forming transmembrane channels. As the membrane is a vital barrier for cell survival, and the integrity of the membrane is essential for the growth of bacteria, they can kill bacteria. It is thought that bacteria are unlikely to develop resistance to AMPs [9,10].

It is well known that a certain correlation exists between antibacterial activity and hemolysis of AMPs [11]. Generally, the better the antibacterial activity, the greater the cytotoxicity. Therefore, AMPs with good antibacterial activity and low cytotoxicity to replace traditional antibiotics must be urgently found [12]. Some reports stated that the whole hemoglobin molecule had no antibacterial activity, but some hemoglobin fragments showed antibacterial activity [13,14]. Previously, our team isolated P3 from the hemoglobin α-subunit of bovine erythrocytes [15], and this study examines the antibacterial activity of P3 analogue HJH-1. By observing the effects of HJH-1 on the morphology of bacteria and measuring the change in the transmembrane potential of bacteria incubated with HJH-1, the membrane-disruption mechanism of HJH-1 is illuminated.

## 2. Results

### 2.1. Peptide Synthesis and Purification

**Figure 1:**
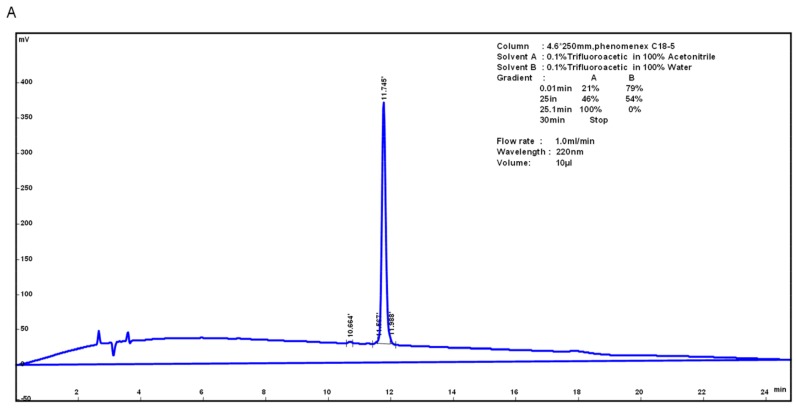

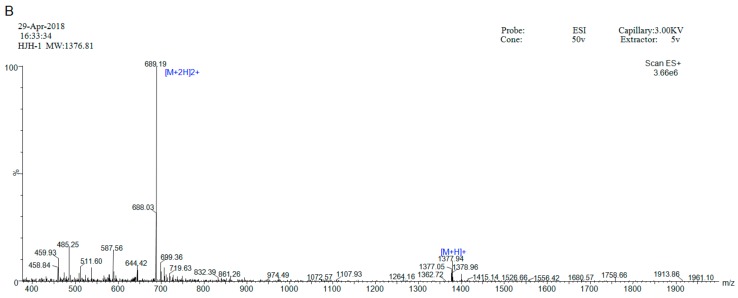
Characterization of AMP HJH-1: (**A**) purification of AMP HJH-1; and (**B**) electrospray ionization (ESI) mass spectrometer (Waters ZQ2000) of HJH-1.

HJH-1 (KLLKHKLLVTLA) contains 12 amino acid residues, and its theoretical isoelectric point was 10.30. The total number of positively charged residues (Arg + Lys) was 3. The aliphatic index was 195.00 and its Grand average of hydropathicity (GRAVY) was 0.783. These results suggest that HJH-1 should be stable. HJH-1 was synthesized to 95% purity as indicated by reverse phase higher performance liquid chromatography (RP-HPLC) purity (Figure 1A). The mass spectrometry data confirmed the identity of HJH-1 as it showed major peaks in the +2 and +1 charge state of 689.19 Da and 13,773.94 Da, respectively (Figure 1B).

### 2.2. Antibacterial Activity

HJH-1 proved to be a highly active antimicrobial agent against all the studied bacterial strains. It was able to inhibit the growth of control strains of Gram-positive, Gram-negative, fungus, and bacteria within a range of 6.25 to 50 µg/mL. Additionally, it inhibited the growth of clinically isolated resistant strains within a range of 6.25 to 25 µg/mL. Clinical bacteria are resistant to three or more of the following antibiotics: gentamicin, amikacin, piperacillin, levofloxacin, imipenem, and colistin. The MIC values of HJH-1 against the bacterial strains are summarized in Table 1.

### 2.3. Stability of HJH-1

**Figure 2:**
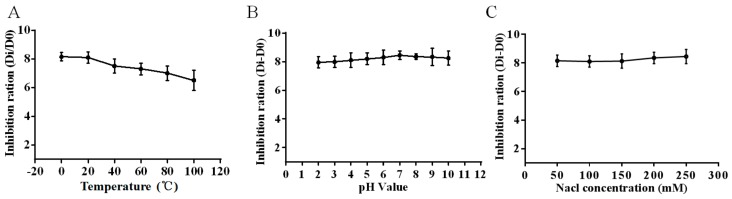
Stability detection of antimicrobial peptide HJH-1. (**A**) Thermal stability test. (**B**) pH stability test. (**C**) Ionic strength stability test. All data are presented as the mean ± SD (*n* = 3).

As shown in Figure 2A, the HJH-1 maintained their antibacterial activities against *E. coli* at the tested temperatures for 30 min. Although the antibacterial activity decreased with increasing temperature, the HJH-1 still had better antibacterial activity after 30 min treatment at 100 °C. The results indicated that HJH-1 has good thermal stability.

As shown in Figure 2B, we observed that the antimicrobial activities of HJH-1 against *E. coli* was not influenced by the medium in the pH range of 4 to 10, and HJH-1 had the best activity when the pH value was 7.

As shown in Figure 2C, the HJH-1 was only slightly weakened in the NaCl solution greater than 0.9% (*w*/*v*, 154 mM) or less, which showed that the ionic concentration had no obvious effect on the HJH-1. 

### 2.4. Toxicity of HJH-1

**Figure 3:**
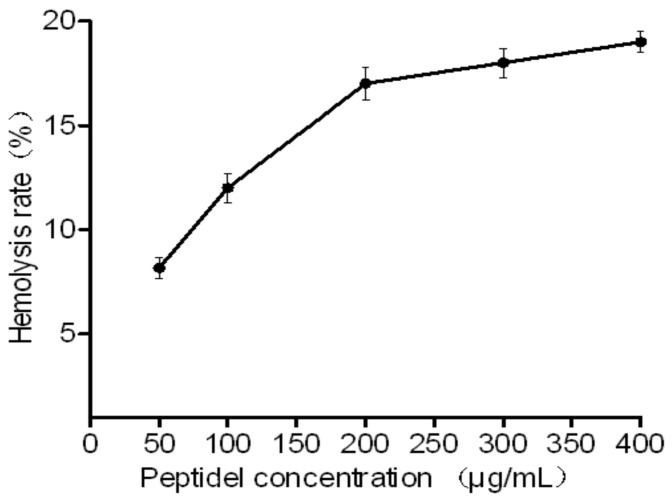
Toxicity of HJH-1 in vitro. Hemolytic activities of HJH-1 at various concentrations upon incubation with red blood cells for one hour. All data are presented as the mean ± SD (*n* = 3).

Because the peptides are promising novel antimicrobial agents, their hemolytic capacities were assessed. The HJH-1 tested exhibited low hemolytic activities against rabbit red blood cells. Even at 400 μg/mL (>5× MIC) for one hour, peptide caused less than 20% hemolysis (Figure 3).

### 2.5. Possible Membrane Activity Mechanism of HJH-1

**Figure 4:**
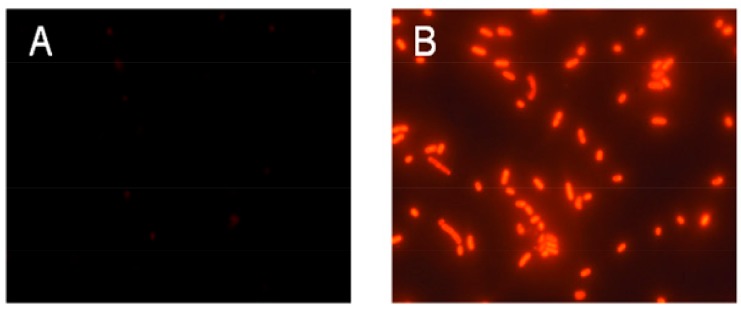
Effect of HJH-1 on *Escherichia coli* membrane permeability. (**A**) *E. coli* was incubated with phosphate buffer saline (PBS) PBS (control) for 30 min. (**B**) *E. coli* was incubated with HJH-1 (1× MIC) for 30 min.

Permeabilization of bacterial cytoplasmic membranes by peptides was investigated by measuring bacterial propidium iodide (PI) uptake. PI only penetrates damaged membranes, staining DNA red. As shown in Figure 4A, after incubation of *E. coli* in logarithmic growth phase with PI at 37 °C for 30 min, PI could not penetrate the cytoplasmic membrane to bind to nucleic acid, and no fluorescence was observed in the field of vision. As shown in Figure 4B, after incubation with *E. coli* treated by HJH-1 with PI using the same method, PI penetrated the cytoplasmic membrane, creating red fluorescence. The results suggested that HJH-1 could damage membranes of *E. coli.*

**Figure 5:**
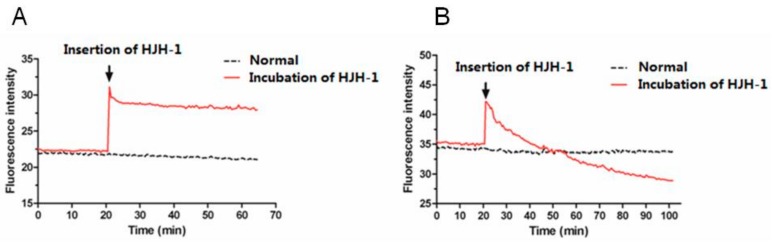
Monitoring of DiBAC4(3). (**A**) Membrane potential of *E. coli* incubated with HJH-1 (1× MIC). (**B**) Membrane potential of red blood cell incubated with HJH-1 (1× MIC).

After DiBAC4(3) diffuses into the cytoplasm of bacteria, it fluoresces. DiBAC4(3) is a robust indicator of the cell membrane potential [16,17], and in the present study, a 10% increase in the fluorescence intensity corresponded to 5 mV depolarisation in the membrane potential of a cell over the range of −20 to −70 mV. When HJH-1 (1× MIC) was added, the fluorescence intensity increased immediately in *E. coli*, as the membranes depolarised rapidly (Figure 5). In comparison, HJH-1 caused the erythrocyte membrane to slowly hyperpolarise.

**Figure 6:**
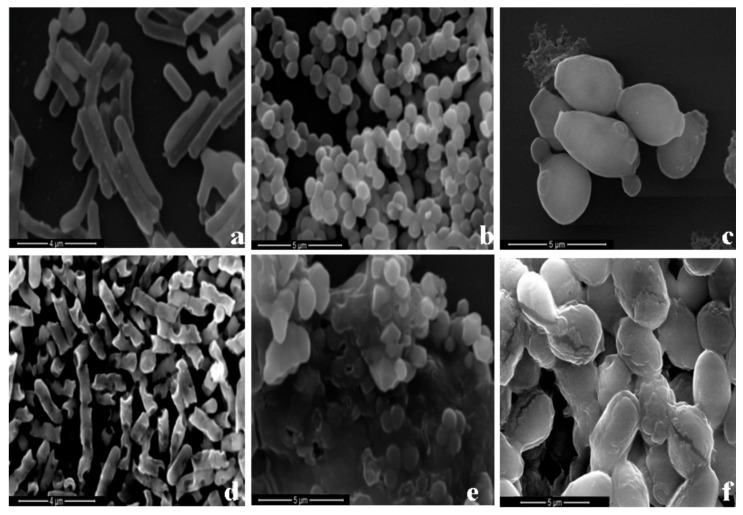
Scanning electron micrograph of 1 × 10^8^ CFU/mL bacteria exposed to HJH-1 (1× MIC). (**a**) Normal *E. coli* control. (**b**) Normal *S. aureus*. (**c**) Normal *C. albican*. (**d**) *E. coli* exposed to HJH-1. (**e**) *S. aureus* exposed to HJH-1. (**f**) *C. albican* exposed to HJH-1.

The effects of HJH-1 at 1× MIC on the morphology of *E. coli*, *S. aureus*, and *C. albicans* were observed with SEM. HJH-1 penetrated the membrane of *E. coli*, forming many collapses (Figure 6a,d). In the presence of HJH-1, the *S. aureus* cells were damaged and slumped (Figure 6b,e). The surface of *C. albicans* became rough. In addition, HJH-1 induced bacterial cell rupture (Figure 6c,f).

**Figure 7:**
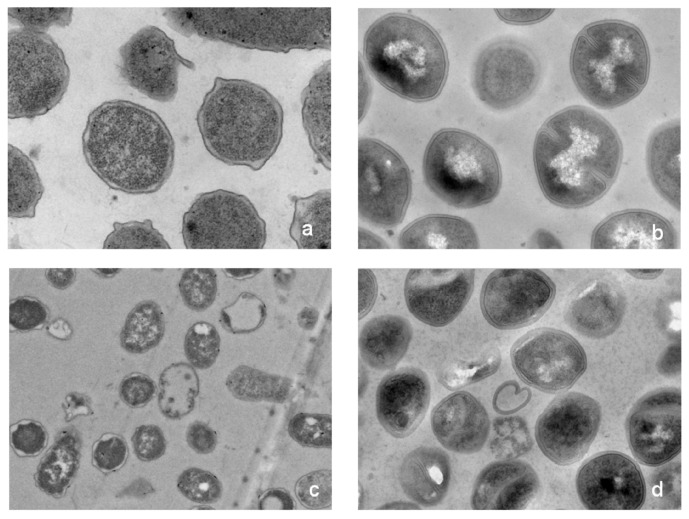
Transmission electron micrographs (TEM) of *E. coli* and *S. aureus*. Bacteria in mid-logarithmic growth were treated with peptides at 1× MIC for three hours. (**a**) Normal *E. coli* control. (**b**) Normal *S.aureus*. (**c**) *E. coli* exposed to HJH-1. (**d**) *S. aureus* exposed to HJH-1.

As shown in Figure 7, transmission electron micrograph (TEM) analysis indicated that the morphology of the bacterial treated with HJH-1 was altered dramatically. The *E. coli* and *S. aureus* in the control without HJH-1 treatment presented a smooth and integral cell surface and dense internal structure. After treatment with HJH-1 at the MIC for three hours, the significant rupture of *E. coli* and *S. aureus* cell membranes (especially the inner membrane or cytomembrane) and the release of cellular contents were observed. The action sites of HJH-1 on the inner membrane may be extensive, and the action sites of HJH-1 on the outer membrane (cytoderm) may be specific.

## 3. Discussion

AMPs are candidate alternatives to conventional antibiotics [18]. However, the molecular weights of many natural AMPs are large and associated with high synthetic costs, which limit their clinical application. HJH-1 is an alkaline peptide with a molecular weight of 1.38 kDa, containing 12 amino acids. In this study, the HJH-1 was synthesized in our laboratory, which will improve the cost.

Currently, more than 2000 antimicrobial peptides have been found. To determine whether an antimicrobial peptide is a medicinal prospect, antimicrobial effects and cytotoxic results must be determined. In this paper, we compared the antimicrobial activity and cytotoxicity of the previously reported human antimicrobial peptides LL-37, pig antimicrobial peptides protegrin 1 (PG-11), PMAP-23, porcine lactoferricin (LFP-20), bovine source AMPs indolicidin and bovine lactoferricin-11 (LFB-11), frog AMPs palustrin-OG1 (OG1), and snake source AMP cathelicidin-Bungarus fasciatus (C-BF), and insect-derived AMP Cecropin PI (CPI) in vitro. The results showed that PG-1, C-BF, and CP1 had the best antimicrobial effect on Gram-negative bacteria, with the MIC range from 1 to 16 μg/mL. PG-1, indolicidin, and C-BF demonstrated the best antimicrobial effect on Gram-positive bacteria, within the MIC range from 2 to 16 μg/mL. In terms of safety, when the concentration of AMPs is within 4 to 256 μg/mL, LFB-11 and LFP-20 have no hemolysis rate, no toxic effect on PBMCS of pigs, and no inhibition on its growth. PMAP23, CP1, and C-BF have low hemolysis rates and cytotoxicity. However, LL37, PG-1, indolicidin, and OG1 had higher hemolysis rates, and significantly inhibited the proliferation of PBMCS [19,20,21,22]. In addition, the potent antimicrobial activity, good stability, and low cytotoxicity of HJH-1 are encouraging. These results indicate that HJH-1 is an excellent candidate for development as a new antimicrobial agent.

Microbial pathogens are ubiquitous in the environment. They occupy a diverse variety of tissues given the range of host immune system. Therefore, it is unrealistic to expect that AMPs will resist all microbial pathogens. The actual mechanisms of the effects of AMPs on micro-organisms are diverse: some form pores, whereas others inhibit bacterial metabolism.

HJH-1 causes serious damage to bacterial cell membranes as shown on SEM. An alignment analysis of the polypeptide chain showed that HJH-1 has high homology with human and pig amino acids in the same region of hemoglobin α-subunit, and these sequences are active against microorganisms [14,23]. The antibacterial activity of AMPs is related to their electrostatic interactions with bacteria, and their hemolytic activity is positively correlated with the hydrophobic interaction of AMPs with the erythrocyte membrane [24]. The positive charge might result in folding and self-assembly of the peptide chain, which produces a pressure effect on the microbial membrane [11,25]. Part of the transmembrane glycoprotein in the red cell membrane contains many hydrophobic amino acids. This hydrophobic region can bind strongly to hydrophobic groups via hydrophobic association. Nevertheless, the HJH-1 peptide chain tends, overall, to be hydrophilic. Consequently, the coupling of HJH-1 oligomers and the red blood cell membrane is weak. This might be why HJH-1 has low toxicity to red blood cells [24]. This partly illustrates the different effects of HJH-1 on bacteria and eukaryotic cells.

In this study, HJH-1 showed a weak hemolytic effect, good stability, and potent antimicrobial activity by disrupting the integrity of the bacterial membrane. However, advanced research on the effects of HJH-1 are needed, such as modifying its sequence to enhance its antibacterial activity and clarify its membrane selectivity mechanism.

## 4. Materials and Methods

### 4.1. Peptide Synthesis

HJH-1 (KLLKHKLLVTLA) was prepared via solid-phase synthesis using 9-fluorenylmethoxycarbonyl (F-moc) chemistry by Tribute polypeptide synthesizer (Proteintech Group, Inc., Cook County, IL, USA), according to the manufacturer’s protocol [26]. Peptides were purified with the aid of RP-HPLC. The purity was 95%. Atomic masses were confirmed using an ESI mass spectrometer (Waters ZQ2000, Milford, MA, USA).

### 4.2. Determination of the Minimum Inhibitory Concentration (MIC)

The tested strains, *Escherichia coli* ATCC25922, *Salmonella pullorum* CVCC3533, *Staphylococcus aureus* ATCC29213, and *Candida albicans* ATCC90029, were bought from Nanjing Bianzhen Biotechnology Co. Ltd. (Nangjing, China). Clinical *Escherichia coli*, clinical *Staphylococcus aureus,* and clinical *Salmonella* were isolated from clinical samples, and they were frozen in the preventive veterinary laboratory of the Henan Institute of Science and Technology. Resistance of these clinical bacteria has been demonstrated. The bacteria were incubated at 37 °C in tryptic soy broth (TSB) (*E. coli* and *S. aureus*) or Sabouraud medium (*C. albicans*), harvested during the exponential phase (OD_600_ approximately equal to 0.6) by centrifugation at 6000× *g* for 15 min, and washed with saline (0.15 M NaCl). Bacteria were resuspended in Muller Hinton (MH) broth at a concentration of approximately 2 × 10^6^ CFU/mL and plated into 96-well plates (50 μL/well) in triplicate. Peptides were dissolved in sterile water at concentration of 4–500 μg/mL and added to each well (50 μL/well). After 24 h of incubation at 37 °C, the minimal peptide concentration affording 100% inhibition was defined as the MIC.

### 4.3. Haemolytic Activity of HJH-1

The hemolytic activity of HJH-1 was measured using rabbit erythrocytes. Blood was collected from ear veins and centrifuged at 800× *g* for 5 min. The supernatant was removed. The packed red cells were washed thrice with saline until the supernatant was transparent. HJH-1 solution (0.5 and 1.0 mg/mL) was mixed with a 5% (*v*/*v*) erythrocyte suspension and incubated for 1 h at 37 °C. As a positive control (100% hemolysis), erythrocytes were lysed with 1% Triton X-100, whereas the negative control (0% hemolysis) comprised erythrocytes suspended in saline. The hemolytic activity was calculated as (OD_peptide_ − OD_negative control_)/(OD_positive control_ − OD_negative control_), measuring the absorbance of hemoglobin in the supernatant at 414 nm [27].

### 4.4. Agarose Diffusion Assay of the Stability of HJH-1

*E. coli* was used as the test strain, and the antibacterial peptide HJH-1 of 1 mg/mL was selected to test the thermal stability, pH value stability, and ionic strength. The activity of HJH-1 with different treatments were determined using a agarose diffusion assay, as previously described, with minor modifications (17). To determine thermostability, the HJH-1 was incubated at 20, 40, 60, 80, or 100 °C for 20 min. For pH value stability, the HJH-1 was incubated in Luria-Bertani (LB) solution with a pH value of 2.0, 3.0, 4.0, 5.0, 6.0, 7.0, 8.0, 9.0, and 10.0 for 20 min. For ionic strength stability, the HJH-1 was incubated in 250, 200, 150, 100, and 50 mM NaCl solution for 20 min.

The initial diameter (D_o_) of all samples (*E. coli*) was measured using Vernier callipers immediately before adding the spread agarose. After 24 h incubation, the diameter (D_i_) of any zone of growth inhibition was measured using Vernier callipers. The control was polymyxin. The inhibition ratio (D_i_–D_o_) reflects the thermostability of HJH-1.

### 4.5. Bacterial Membrane Permeability Assay

Membrane permeability was determined by measuring bacterial uptake of propidium iodide (PI). *E. coli* cells were incubated with 50 μg/mL HJH-1 at 37 °C for 30 min, whereas the control was incubated with PBS alone. Next, PI (100 μg/mL) was added to all the samples, and the suspensions were incubated at 37 °C for 10 min. Finally, samples were examined under a fluorescence microscope (Eclipse 80i; Nikon, Japan).

### 4.6. Measuring Membrane Potential

The anionic membrane potential probe *bis*-(1,3-dibutylbarbituric acid) trimethine oxonol [DiBAC_4_(3)] is a *bis*-barbituric acid oxonol dye with an excitation maximum of ~490 nm. It has been used to measure the effects of antibiotics on the membrane potential of plasma membrane cells. Red blood cells or bacteria were incubated with 5 µmol/L DiBAC_4_(3) in 4-(2-Hydroxyethyl)piperazine-1-ethanesulfonic acid (HEPES) solution for 30 min at 37 °C. The peptide was added to the suspension automatically at various stages during the experiments. The fluorescence intensity data were collected using Varioskan Flash at 30 s intervals.

### 4.7. Effect of HJH-1 on Bacterial Morphology

Washed bacterial cultures (1 × 10^6^ CFU/mL) were incubated with HJH-1 for 6 h in 10 mM phosphate buffer and subject to three-step centrifugation (5000× *g* for 10 min). The bacteria were resuspended in 10 mM phosphate buffer (pH 7.4) and fixed using 5% (*v*/*v*) glutaraldehyde for 3 h. Bacteria were added drop-wise to an 8 mm coverslip, which was dehydrated sequentially for 15 min each in 60, 70, 80, 90, and then 100% (*v*/*v*) ethanol twice. Then the coverslip was freeze-dried in an ultra-low-temperature freeze dryer. The coverslips were mounted on aluminum stubs, sputter-coated with a fine coating of gold, and observed using scanning electron microscopy (SEM, Quanta 200, FEI Company, Hillsboro, OR, USA).

Peptides (1× MIC) were incubated with *E. coli* (1 × 10^6^ CFU/mL) and *S. aureus* (1 × 10^6^ CFU/mL) for 3 h in a total volume of 1 mL in buffer, respectively. After fixed, dehydrated, and embedded, the specimen sections were stained by uranylacetate and alkaline lead citrate for 15 min, respectively and observed in transmission electron microscope (TEM), Model HT 7700 (HITACHI, Tokyo, Japan).

## 5. Conclusions

In summary, this study screened AMP HJH-1, which has broad spectrum activity and good stability, without hemolysis activity. HJH-1 plays its antibacterial role by changing the membrane potential and destroying the integrity of the membrane. These results suggest that HJH-1 may be feasible and promising to develop as a potent antimicrobial agent for the food or animal feed additive industry.

## Figures and Tables

**Table 1:** Summary of the minimum inhibitory concentration (MIC) values of HJH-1 against all tested bacterial strains. Results represent triplicates.

Bacterial Strain	Bacterial Number	MIC ^a^ (µg/mL)	
		P3	Amp
*Escherichia coli*	ATCC25922	12.5	0.5
Clinical *Escherichia coli* ^b^	None	25	>200
*Salmonella pullorum*	CVCC3533	6.25	0.5
Clinical *Salmonella* ^b^	None	6.25	>200
*Staphylococcus aureus*	ATCC29213	25	0.5
Clinical *Staphylococcus aureus* ^b^	None	25	>200
*Candida albicans*	ATCC90029	50	-

^a^ A minimal level of 99.9% inhibition is indicated. > denotes no activity detected at the concentration indicated. - denotes not assayed. ^b^ Resistant to three or more of the following antibiotics: gentamicin, amikacin, piperacillin, levofloxacin, imipenem, and colistin.

## References

[1] Fischbach M.A., Walsh C.T. (2009). Antibiotics for emerging pathogens. Science.

[2] Kumarasamy K.K., Toleman M.A., Walsh T.R., Bagaria J., Butt F., Balakrishnan R., Chaudhary U., Doumith M., Giske C.G., Irfan S. (2010). Emergence of a new antibiotic resistance mechanism in India, Pakistan, and the UK: A molecular, biological, and epidemiological study. Lancet Infect. Dis..

[3] Moellering R.C. (2010). NDM-1—A cause for worldwide concern. N. Engl. J. Med..

[4] Adegoke A.A., Faleye A.C., Singh G., Stenstrom T.A. (2016). Antibiotic Resistant Superbugs: Assessment of the Interrelationship of Occurrence in Clinical Settings and Environmental Niches. Molecules.

[5] Hood J.L., Jallouk A.P., Campbell N., Ratner L., Wickline S.A. (2013). Cytolytic nanoparticles attenuate HIV-1 infectivity. Antivir. Ther..

[6] Brown K.L., Hancock R.E. (2006). Cationic host defense (antimicrobial) peptides. Curr. Opin. Immunol..

[7] Chen Y., Guarnieri M.T., Vasil A.I., Vasil M.L., Mant C.T., Hodges R.S. (2007). Role of peptide hydrophobicity in the mechanism of action of α-helical antimicrobial peptides. Antimicrob. Agents Chemother..

[8] Silva T., Gomes M.S. (2017). Immuno-Stimulatory Peptides as a Potential Adjunct Therapy against Intra-Macrophagic Pathogens. Molecules.

[9] Rathinakumar R., Walkenhorst W.F., Wimley W.C. (2009). Broad-spectrum antimicrobial peptides by rational combinatorial design and high-throughput screening: The importance of interfacial activity. J. Am. Chem. Soc..

[10] Toke O. (2005). Antimicrobial peptides: New candidates in the fight against bacterial infections. Pept. Sci..

[11] Hou J., Liu Z., Cao S., Wang H., Jiang C., Hussain M.A., Pang S. (2018). Broad-Spectrum Antimicrobial Activity and Low Cytotoxicity against Human Cells of a Peptide Derived from Bovine alphaS1-Casein. Molecules.

[12] Kohn E.M., Shirley D.J., Arotsky L., Picciano A.M., Ridgway Z., Urban M.W., Carone B.R., Caputo G.A. (2018). Role of Cationic Side Chains in the Antimicrobial Activity of C18G. Molecules.

[13] Nedjar-Arroume N., Dubois-Delval V., Adje E.Y., Traisnel J., Krier F., Mary P., Kouach M., Briand G., Guillochon D. (2008). Bovine hemoglobin: An attractive source of antibacterial peptides. Peptides.

[14] Liepke C., Baxmann S., Heine C., Breithaupt N., Ständker L., Forssmann W.-G. (2003). Human hemoglobin-derived peptides exhibit antimicrobial activity: A class of host defense peptides. J. Chromatogr. B.

[15] Hu J., Xu M., Hang B., Wang L., Wang Q., Chen J., Song T., Fu D., Wang Z., Wang S. (2011). Isolation and characterization of an antimicrobial peptide from bovine hemoglobin α-subunit. World J. Microbiol. Biotechn..

[16] Yamada A., Gaja N., Ohya S., Muraki K., Narita H., Ohwada T., Imaizumi Y. (2001). Usefulness and Limitation of DiBAC4(3), a Voltage-Sensitive Fluorescent Dye, for the Measurement of Membrane Potentials Regulated by Recombinant Large Conductance Ca2+-Activated K+ Channels in HEK293 Cells. Jpn. J. Pharmacol..

[17] Kralj J.M., Hochbaum D.R., Douglass A.D., Cohen A.E. (2011). Electrical spiking in Escherichia coli probed with a fluorescent voltage-indicating protein. Science.

[18] Nagarajan D., Nagarajan T., Roy N., Kulkarni O., Ravichandran S., Mishra M., Chakravortty D., Chandra N. (2018). Computational antimicrobial peptide design and evaluation against multidrug-resistant clinical isolates of bacteria. J. Biolog. Chem..

[19] Mojsoska B., Jenssen H. (2015). Peptides and Peptidomimetics for Antimicrobial Drug Design. Pharmaceuticals.

[20] Tjabringa G.S., Rabe K.F., Hiemstra P.S. (2005). The human cathelicidin LL-37: A multifunctional peptide involved in infection and inflammation in the lung. Pulm. Pharmacol. Ther..

[21] Cole A.M., Waring A.J. (2002). The role of defensins in lung biology and therapy. Am. J. Respir. Crit. Care Med..

[22] Falla T.J., Karunaratne D.N., Hancock R.E. (1996). Mode of action of the antimicrobial peptide indolicidin. J. Biolog. Chem..

[23] Sun Q., Shen H., Luo Y. (2011). Antioxidant activity of hydrolysates and peptide fractions derived from porcine hemoglobin. J. Food Sci. Technol..

[24] Brogden K.A. (2005). Antimicrobial peptides: Pore formers or metabolic inhibitors in bacteria?. Nat. Rev. Microbiol..

[25] Mani R., Cady S.D., Tang M., Waring A.J., Lehrer R.I., Hong M. (2006). Membrane-dependent oligomeric structure and pore formation of a β-hairpin antimicrobial peptide in lipid bilayers from solid-state NMR. Proc. Natl. Acad. Sci. USA.

[26] Karas J., Sani M.-A., Separovic F. (2017). Chemical Synthesis and Characterization of an Equinatoxin II(1–85) Analogue. Molecules.

[27] Norwitz E.R., Tsen L.C., Park J.S., Fitzpatrick P.A., Dorfman D.M., Saade G.R., Buhimschi C.S., Buhimschi I.A. (2005). Discriminatory proteomic biomarker analysis identifies free hemoglobin in the cerebrospinal fluid of women with severe preeclampsia. Am. J. Obstet. Gynecol..

